# A Geometrical Perspective for the Bargaining Problem

**DOI:** 10.1371/journal.pone.0010331

**Published:** 2010-04-26

**Authors:** Kelvin Kian Loong Wong

**Affiliations:** School of Aerospace, Mechanical and Manufacturing Engineering, RMIT University, Bundoora, Australia; University of East Piedmont, Italy

## Abstract

A new treatment to determine the Pareto-optimal outcome for a non-zero-sum game is presented. An equilibrium point for any game is defined here as a set of strategy choices for the players, such that no change in the choice of any single player will increase the overall payoff of all the players. Determining equilibrium for multi-player games is a complex problem. An intuitive conceptual tool for reducing the complexity, via the idea of spatially representing strategy options in the bargaining problem is proposed. Based on this geometry, an equilibrium condition is established such that the product of their gains over what each receives is maximal. The geometrical analysis of a cooperative bargaining game provides an example for solving multi-player and non-zero-sum games efficiently.

## Introduction

### 1.1 Scope and Objective

Pareto-optimality is concerned with the logical analysis of optimizing a set of strategies in a real life situation involving the interaction of more than one individual, that can be mathematically modeled as a game [1],[2]. The payoff in a game can be non-measurable psychological parameters such as *utility*, prestige, desire, security level, or measurable quantities that serve as a common medium of exchange. Some simple games, such as the bargaining problem [3], are known as non-zero-sum games and the players try to achieve a win-win situation. The game “paper-rock-scissors” is a zero-sum game, since the payoff to the winner of a single instance of the game is equal to the loss of the other player. In any game, there exist choices of strategies that may be adopted to maximize sum total payoff. For a generic *n*-player game, there is always a solution for the optimal strategy for each person. This strategy guarantees their average payoff or loss is maximized or minimized under the assumption that the opposing player also uses an optimal strategy. This solution corresponds to the equilibrium point (EP) of the game, and may be non-deterministic, in that the optimal choice of strategy to use in any given play of the game is chosen randomly according to a probability distribution over all possible strategy choices.

In this paper, the focus is on the analysis of non-zero-sum games. Another classic non-zero-sum game is “the Prisoner's Dilemma” [4],[5],[6] but it pertains to a non-cooperative one. More practical games typically involve multiple players in areas of economics [7] and mathematical biology [8],[9],[10]. The computation of EP increases significantly in complexity for multi-player, multi-strategy and non-zero-sum cooperative games [11].

The notion of an EP is the key ingredient in a game, and is what we aim to obtain. There may be more than one EP; and for a two-person zero-sum game, it is simply the set of all pairs of opposing good strategies [12] whereas for a non-zero-sum game, it consists of pairs of reinforcing good strategies. The approach to solving non-zero-sum cooperative games, for the case of two players, can be better understood if the *utility* gains of all possible actions are computed and plotted as a set of alternatives on a two-dimensional graph. The convex point at the vertex of the possible set of solutions corresponds to the optimal *utility* gain for both persons. For an *n*-players game, this treatment can be extrapolated to a set of alternatives on a multi-dimensional graph.

### 1.2 Theory of Utility

The theory of *utility* can be observed in many games. In the simplest bargaining scenario presented by Nash, two cooperative individuals have a certain list of goods to barter trade [12]. This simple example can be extended to other situations of bargaining, such as employer and union negotiation, or to that of two villages with different resources and an aim of optimizing economic benefit in trade. As an example, we present a primitive scenario whereby two villages, hereby labeled as Villages 1 and 2, are involved in barter trading of specific goods. Village 1 relies on coal production as the main source of its revenue. Village 2 is assumed to have abundant cattle production but has insufficient coal resources. We make a further assumption that cattle are of lesser abundance, and hence these goods enjoy a higher value compared to coal. If the two villages concentrate on their strengths of production individually, and perform trading of the goods that have different *utilities* to each party, a point of equilibrium in trade will be reached at a certain point of time. Coal will naturally be more valued within Village 2 due to limitation, but a huge quantity of cattle will essentially not be produced if greater efforts are aimed at producing coal. It is better to trade cattle for coal since this is a less expensive alternative in terms of value that pertains to the good. Village 1 that trades coal for cattle perceives and thinks likewise. A certain equilibrium trading quantity of the two goods can be reached in a mutual trade agreement depending on the *utility* value of goods to each village. This equilibrium corresponds to the payoff of the two villages.

From a psychological perspective, the *utility* value of an item to an individual is dictated by state of mind at a particular time. It varies according to the events that occurred to the person previously, and is evidently a random psychological parameter. Analogously, the security level of performing a specific task can also be linked to this *utility* value. In practice, we are all dictated by this value in determining our actions or performing any type of task assigned to us. For instance, a shopper may decide to buy a tin of cookies instead of a box of chocolates because the cookies appeal more to that person in terms of taste, price or aesthetic property, and hence, has a higher *utility* value in comparison. This *utility* value may change when the shopper tires of eating cookies after a few days and desires a taste of chocolate the next time. Desire or *utility* of a grocery to a shopper may also vary based on available quantity at its time of acquisition.

It is difficult to construct a perfect model of a game that is a reflection of reality. There will always be numerous outcomes to consider even for the simplest games. For example, when volumes of items are divided for exchange, its value to each player may change. There are usually too many variables such as the bargaining abilities of the players, the norms of the society, and the variation in utility of the items over time for the formal theory to be accommodated [14]. But understanding bargaining games from a simplistic perspective can assist in the study and formulation of frameworks for determining EP.

### 1.3 The Bargaining Problem

For the bargaining problem, we illustrate how, as a special case, the two persons can perform barter trade such that their *utility* gains are maximized. We assume two players - Player 1 and Player 2 who are in a position to barter goods but have no money to facilitate the exchange [12]. Bargaining theory is a generalized concept of the two-person bargaining problem. The game is a cooperative one as both players have complete information about the game; each player is fully aware of the payoff or profit for themselves and their co-players, for every possible transaction. Such games are also known as *cooperative games* in which all players have identical interests.

In the two-player bargaining situation, a compact convex metrical space *S_i_* of mixed strategies *σ_i_* pertains to Player *i*, for 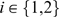. These mixed strategies represent the courses of action the player can take independently of the other players. The randomization process of establishing all possible strategy alternatives illustrates the possible joint courses of action by the players. This set of alternatives can be represented by a convex polytope in the plane with the dimensions of *utility* gains for the players. For each pair of mixed strategies (*σ*_1_, *σ*_2_) from (*S*_1_, *S*_2_), the payoffs for the deployment of these strategies are denoted by Π_1_(*σ*_1_, *σ*_2_) and Π_2_(*σ*_1_, *σ*_2_) respectively. Such payoff of each mixed strategy pair corresponds to a point in the convex polytope of the super set strategy alternatives [13].

An outcome is in equilibrium if there is no other possible agreement that allows both players to have higher payoffs simultaneously [14]. The barter trade such that maximum *utility* gain *G_i_* is achieved is known as Pareto-optimal. Note that gain is the excess of payoff after bargaining over the initial payoff before strategy choices are chosen. Nash has shown that obtaining the maximum of the product of the two *utility* gains (*G*_1_ and *G*_2_) from the set of alternatives, known as the Nash product *G*_1_ *G*_2_, will attain the Pareto-optimal solution for the bargaining situation. Pareto-optimality is a non-zero sum Nash game equilibrium point that determines the Pareto efficiency of the outcome. It is also worthwhile mentioning that there may be more than one equilibrium point, and this set of points can be defined as the equilibrium point (EP).

**Figure 1:**
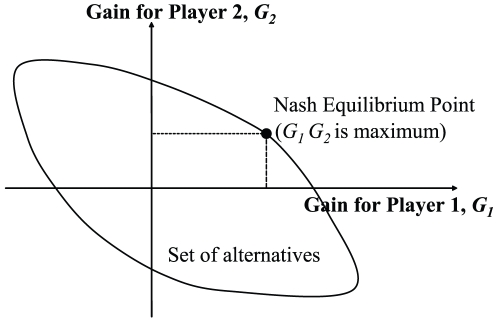
Set of bargaining alternatives for two players whereby the Nash equilibrium corresponds to optimal *utility* gains.

The combinatorial plot for this bargaining situation is illustrated in Figure 1, where the set of alternatives for all possible item exchanges is enclosed by a boundary curve. We make the assumption that at least one item is possessed by each player in the end. The super set of alternatives results in a convex polygon whereby the product of maximum *utility* gains is maximized at its vertex. In practice, we aim to optimize the Nash product *G_1_G_2_*.

The solution is a Pareto-optimal outcome, in which the joint profits by all parties are maximized. It may be worthwhile noting that the Nash equilibrium is not unique as multiple or even an infinite number of strategies that pertain to the Nash equilibria exist. In this game theoretical setup, all items are discrete, and the discreteness of the payoffs increases the complexity of attaining the solution.

### 1.4 Pareto-optimal Equilibrium

Based on a multi-player bargaining situation, establishing the payoff matrix containing the super set of strategy choice alternatives is computationally expensive. It is to be noted that proving the existence of the Pareto-optimal equilibrium point and finding the solution set at the equilibrium are different tasks. In general, computation of a Pareto-optimal equilibrium point in mixed strategies of a finite game poses a numerical challenge for the following reason. For multiple players, determining the Pareto-optimal equilibrium in mixed strategies amounts to solving a system of multivariate polynomial equations of high order [13] and, as a rule, does not have an explicit solution. Despite significant progress in recent years, algorithms for computing equilibrium are still not competent at solving very large games. Approximate solution methods are often the best computed solution or EP set. This forms the problem definition in our study.

The concept of representing *utility* of strategy executions geometrically is used to answer the fundamental question in the Pareto-optimal equilibrium solution. Obtaining information regarding the strategy options and payoffs to every player, and with respect to other players, is crucial to the computation of the Pareto-optimal EP. This information can be represented spatially in a geometrical framework. It turns out that by using the spatial game setup, the extraction of the subset of strategy alternatives from the superset can be achieved and the convergence to the Pareto-optimal solution using less computation can be obtained. Such a concept forms the main basis of this paper, and we will examine the game theoretical geometry in greater details with the support of case studies.

### 1.5 Representation of Utility as Geometrical Distance

The game theoretical studies by von Neumann and Morgenstern (1980) is based on the representation of all outcomes of *n*-player games as payoff vectors such that points exist in an *n*-dimensional *utility* space [15]. This has been a standard representation of *utility* space for *n*-person game models to date. In the spatial game model that they have created, outcomes of the games have been assumed to lie in some low-dimensional Euclidean space such that *utilities* to the players are defined in terms of distance from their most preferred, or ideal points [16]. Such a representation is useful for establishing outcomes that correspond to public good (all individuals obtaining benefit from the same outcome). The model assumed that *utility* is a decreasing function of the distance between the achieved outcome and the ideal point [17].

For example, their *m*-dimensional spatial game is described as a collection of *n* points *P_i_*, *i* = 1,…,*n*, in *m*-dimensional Euclidean space *R^m^*. Point *P_i_* is player *i*'s ideal point. The convex hull of the points 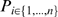 is the Pareto-optimal set. The points of space *R^m^* are the items in the game that we have discussed. The players are to choose among all the items in the game what they wish to own. It is assumed that a player may be most satisfied with an item at *P_i_*, however failing to obtain that, the next possible closest item will be chosen.

The next section will focus on presenting a more refined technique for determining the Pareto-optimality of a bargaining problem for multi-players (*n*>2). The fundamental concepts of representing *utility* as a distance in space will be presented.

## Methods

### 2.1 Geometrical Representation of Utility

Suppose the payoff value to the player of a strategy item being executed can be represented by the “item-to-player” distance, such that items of higher *utility* value have a higher spatial proximity. In effect, the proximity value would be the inverse of the payoff value. For two parties, if one represents all the items based on payoff value on a two-dimensional space, an equilibrium line could be drawn to assign that item to the respective player such that payoff value for both is maximized. This technique removes the need to generate the payoff gain for every single possible set of strategy execution, hence avoiding intense computational load. In fact, this method of assigning items of significance closer to the owner will effectively eliminate the consideration of all the strategy alternatives, based on the fact that the product of gain will be maximal at the location near the equilibrium line. For example, it is not a recommendable strategy to assign items of low payoff to any one player. Now, this distance-based approach is explored to derive the EP of a bargaining situation.

**Figure 2:**
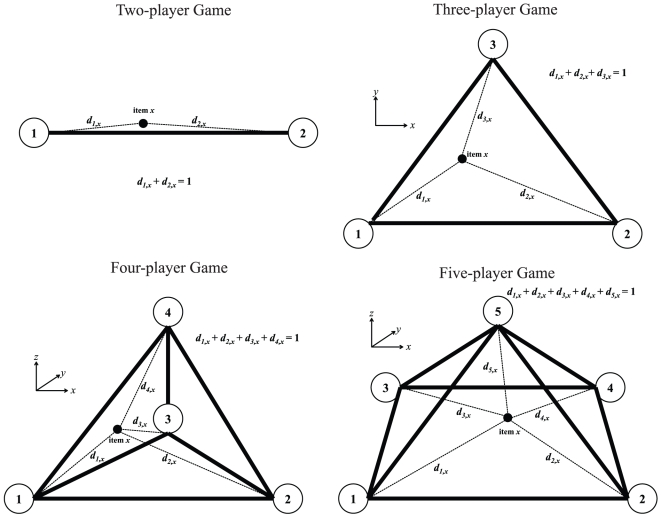
Geometrical distribution of items whose cross-links represent their distances to every person in an *n*-player game.

The convex polytope of a multi-player game can be simplified to a three dimensional spatial representation with the player-to-player distances defined to be constant as shown in Figure 2. In this approach, all items are represented as geometrical points that lie within a boundary or space enclosed by the players (labeled as 1, 2,…, *n*) that are presented as vertices of this spatial volume. For example, based on three players, the items will lie within an enclosed triangle. With four players, the items are enclosed by a tetrahedron with four vertices. Based on a five-player game, the positioning of items is within a space enclosed by a pyramid. In general, *n* number of players will result in an *n*-polyhedron defined by the ideal position of all players representing its vertices. The distance between player-to-player decreases as more players participate because for an item *x*, its normalized and relative distances to all the players (labeled as *d_1,x_*, *d_2,x_*,…, *d_n,x_*) add up to a unitary value. Therefore, the space enclosed by the polyhedron becomes smaller as the number of players increases.

#### 2.1.1 Geometrical Distance of Item

We introduce the following notation:

*u_i,x_* represents the *utility* of item *x* to player *i*;
*d_i,x_* represents the *normalized distance* of item *x* to player *i* with respect to other players;

The distance of an item to the player is defined as a decreasing function of the *utility*. The inverse proportionality function is used. Here, the distance of item *x* to player *i* is defined to be the inverse of *u_i,x_*. Next, based on every item, its *normalized distance* is given by the ratio of distance for player *i* to the sum of distances for all players. The following equation presents the geometrical parameter as
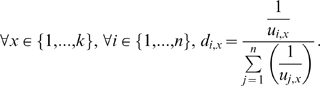
(1)


#### 2.1.2 Prioritized Assignment of Items

The items are arranged spatially based on their *normalized distances* to every player in ascending order. Here, a list of items ordered in terms of *priority* for player *i* is denoted as 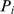 where
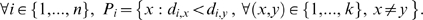
(2)The *normalized distance* contains information regarding the *priority* of the item to one player with respect to the others based on the geometrical treatment presented in Section 3.1.1. The *priority* of an item is an indication of its value or importance to the player, and is the inverse of this *normalized distance*. Therefore, assigned items in terms of *priority* is defined here as a possession list of items in descending order of importance to a player.

Combinatorial analysis for all alternatives is computationally expensive. Assigning items of lower *priorities* to a player shifts the equilibrium away from the Pareto-optimality. On the contrary, assignment of items with higher *priorities* converge the solution set towards an EP. Assignment of items to a player by traversing them from the highest *priority* to the lowest one enhances computational resources. Therefore, instead of using a combinatorial technique to obtain the optimal product of gain for all players, a more efficient assignment approach based on *priority* of items can be applied. Items can be spatially arranged in terms of value for every player in the game. An item *priority* to a player is based on the *normalized distance* with respect to all players.

In summary, the method of assigning items focuses on considering a set of alternatives that lies close to the real solution set. In a way, it eliminates the redundant computation of alternatives that deviates from the EP. In Section 4, we demonstrate that the EP solution set lies within the reduced set of alternatives.

### 2.2 Basis of Convergence towards Equilibrium

It is computationally expensive to consider all bargaining alternatives from a combinatorial set. This section describes a technique for evaluating a bargaining game via item assignment to each player. It analyzes the game based on multiple cases of item assignment by taking into account one player that pertains to each case. For *n* players, such assignment of items is performed *n* times.

The *utility* is related to *priority*. Since *priority* is the inverse of *normalized distance* of a bargain item, a property that relates to the payoff per *priority* is introduced here. To achieve this, we will discuss this indicator from a mechanical perspective. For a mechanical lever system, the moment 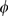 by weight 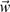 about a point *Q* is defined as the product 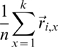 = |
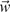
|*d*, where *d* is the (perpendicular) distance between *Q* and the line of action *L* of 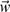. If 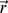 is the vector from Q to any point A on L, then the moment vector of 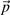 about *Q* is given by 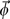 = 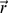
×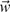.

**Figure 3:**
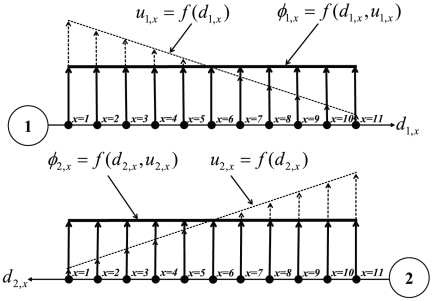
Spatial positions of items presented in *x*-direction and magnitudes of their *utilities* and *utility-distance products* in *y*-direction for two players.

Assume two players (*i* 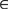
{1,2}) possessing items given by *x* 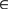
{1,…,11} such that *utility* 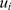 is a function of importance of an item to a player and represented by its *normalized distance* 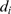 as shown in Figure 3. Here, 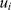 is assumed to be a decreasing function of 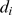. A *utility-distance product* of an item value (which is related to its importance in terms of payoff to the player) is based on distance *d* from a player spatially and in vector form. We denote this entity as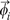, which is a function of 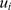 and 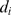. For *n* players, using each player as a pivot point, the importance of an item can be weighing about *n* number of pivots such that their *utility-distance product* vectors have the same magnitude.

**Figure 4:**
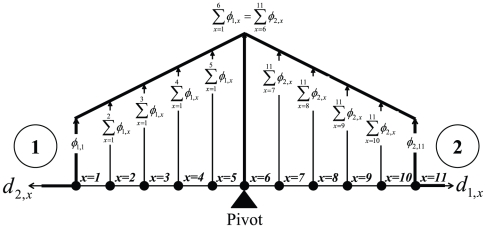
Determination of equilibrium based on distribution of *utility-distance product* vectors about a pivot point for two players.

From a mechanical perspective, equally spaced weights on a lever are aligned along the same direction such that the weights on the left hand side generate a collective moment that opposes the moment caused by the weights on the right. By the same concept, to maintain zero *utility-distance product* equilibrium, the sum of *utility-distance product* vectors by all the items is equally divided for the players (Figure 4). Selecting items of closer distance to the player maximizes their payoffs. Maximizing the sum of *utility-distance products* for every player simultaneously provides the equilibrium solution for such theoretical game using this geometrical setup.

Assume that the items are analogous to equal quantity of weights on a lever system that is balanced such that
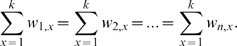
(3)Consider that the *utility-distance product* vector of an item *x* about a player *i* from spatial vector 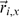 is denoted by 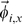. A player has *k* items, each of value 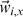. For an ideal condition whereby all items lie in a space between two vertices representing players, and at equal distances. Here, the vector sum of *utility-distance products* is
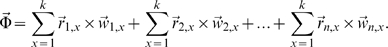
(4)Due to our assumption that *utility* decreases linearly with respect to distance, a pivot positioned at a distance of 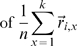 causes the vectors given by 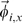 = 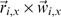 to sum up to zero where
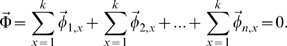
(5)In practice, the vector 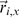 may vary for different items. A more appropriate determination of pivot location can be based on balancing *utility-distance products* vectors with respect to *n* player, which is given by
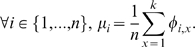
(6)In theory, *utility-distance product* of an item is analogous to moment of force by weights based on a lever system. By suitably locating a pivot location such that distribution of the *utility-distance product* vectors are uniformly positioned about a pivot, equilibrium can be achieved. This concept can effectively reduce computational load of item assignments in a multi-player game theoretical situation. The reduced set of alternatives that is determined is the solution set closer to Pareto-optimality, which forms the basis of convergence towards EP.

#### 2.2.1 Utility-Distance Product of Item

From Section 3.1.1, the properties utilized in our framework are *u_i,x_*, which represents the *utility* of item *x* to player *i*; And *d_i,x_* represents the *normalized distance* of item *x* to player *i* with respect to other players. We also define the *utility-distance product* 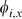 of the item *x* from distance *d_i,x_* for the player *i*.

The *utility-distance product* for an item denoted as *x*, is computed by multiplying the *normalized distance* of that item with its corresponding payoff value for player *i* such that
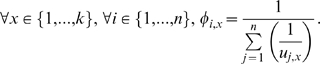
(7)


#### 2.2.2 Pareto-Optimality Based on Geometry

For *k* items, every item *x* is prioritized based on *normalized distances d_i,x_* from player *i* to form a list given by *P_i_* (Equation (2)). Here, *P_i,x_* represents an item *x* in the order of *priority* for a player. Cumulative values of *utility-distance products* are determined based on the list 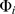, which consists of *k* items in descending order of *priority*. Here, 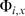 corresponds to an item at position *x* in this list. The possession list of every player is initialized to null. We define 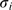 as the items after bargaining in one strategy set for the player *i* respectively. The items are traversed in the high to low *priority* direction, and assigned to player *i*'s possession list until the cumulative *utility-distance products* equals 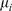. Recall that 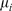 is the quantification of *utility-distance products* up to a point of equilibrium (Equation (6)). The item that corresponds to the pivot location is hereby denoted as 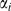. We note that 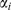 may not lie exactly at the pivoting point. Mathematically,
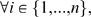


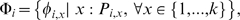
(8)

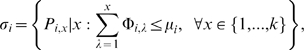
(9)

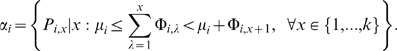
(10)Depending on the nature of the game, different interpretations of an EP can be made. If the items are assumed to be discrete, then assignment of items up to 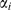 for player *i* forms a strategy set *σ_i_*. Repeating such an assignment for *n* players forms *n* strategy sets, {*σ*_1_, *σ*_2_, …, *σ_n_*}, whereby this *n*-tuple of strategies forms the negotiation set of the game. Pareto-optimal equilibrium is attained when no player can increase their payoff any more by changing strategy and assuming that none of the other players is going to change their strategies.

## Results

### 3.1 Definition of *n*-player Bargaining Case Studies

The multi-player bargaining scenario is a cooperative game; information is shared and every member trades in such a manner that their strategies will mutually benefit all the players in the game. The existence of a transferable commodity is assumed to be present. From the bargaining perspective, the items of transaction are analogous to the strategy choices, and the *utility* gain of a typical transaction is analogous to the payoff of executing a particular strategy. The proposed case studies will only work for relative payoff values for the players. In this paper, we denote the payoff as a function of item *x*, whereby Π*_i_* (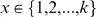) for player *i* and *k* number of items. To limit the EP to only one solution (assuming that no two or more items are similar), the optimality condition is defined in this paper as a function of the product of gains.

We have assumed that the items are being assigned based on non-initialized possession lists. The *utility* gains are positive provided that the initial total payoff value of any one list does not exceed that of the created solution possession list. Assume that the initial payoff for Player *i* is Π*_i_*′. Then for *n* players, the product of payoff gain is given by
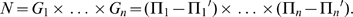
(11)For all players *i* such that Π*_i_* is greater than Π*_i_*′, positive payoff by all persons is achieved. Negative gain situations can be prevented by first assigning the items starting from those with higher *priorities* in the ordered list based on *normalized distances* until their aggregate payoff values equal or exceed the initial payoff value. The remaining items are then assigned again starting from the one with the next highest *priority*.

### 3.2 Two-player Bargaining Game (*n* = 2)

A primitive setup of a two-village bargaining problem is defined here, in which Villages 1 and 2 possess goods to perform barter trade (Table 1), with the objective of maximizing the gains.

**Table 1:** Utility of goods for Villages (i {1, 2}).

Village	*x*	Goods	*u_1,x_*	*u_2,x_*
**1**	1	Coal	2	4
	2	Cattle	2	2
	3	Mineral	2	1
	4	Oil	2	2
	5	Salt	4	1
**2**	6	Iron	10	1
	7	Steel	4	1
	8	Wine	6	2
	9	Gas	2	2

The initial values of the *utility* sums for the villages are
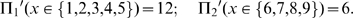
(12)In Table 2, based on Equations (1) and (7), the *normalized distances* and *utility-distance product* for Villages 1 and 2 (denoted as *i* = 1 and 2 respectively) are presented.

**Table 2:** *Utility-distance product* of goods for Villages (*i* 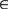
{1, 2}).

*x*	Goods	*d_1,x_*	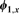	*d_2,x_*	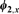
1	Coal	0.667	1.334	0.333	1.334
2	Cattle	0.500	1.000	0.500	1.000
3	Mineral	0.333	0.667	0.667	0.667
4	Oil	0.500	1.000	0.500	1.000
5	Salt	0.200	0.800	0.800	0.800
6	Iron	0.0909	0.909	0.909	0.909
7	Steel	0.200	0.800	0.800	0.800
8	Wine	0.250	1.500	0.750	1.500
9	Gas	0.500	1.000	0.500	1.000

The equilibrium condition is
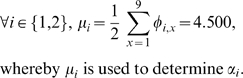
(13)The cumulative *utility-distance products* of the goods are listed in Table 3 in terms of *priority* for Village 1 that is ordered from left to right. For Village 2, the cumulative operation starts in the opposite direction. The geometrical treatment gives 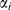 as Mineral (*x* = 3). An EP set is determined here: all goods of higher *priority* to Village 1 up till before 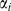 are assigned to this village and the rest of the goods pertain to Village 2. Based on the possession lists of the two players, we obtain
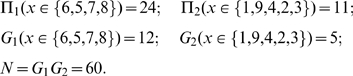
(14)The same scenario in a two-village game is considered but discussions of special situations such as negative *utility* gains are established using a different data set. An example using a data set with a significantly different total *utility* values between the two players can illustrate the problem.

**Table 3:** Cumulative *utility-distance products* of goods for Villages (*i* 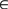
{1, 2}).

Goods assigned to Village 1					Goods assigned to Village 2			
Iron	Salt	Steel	Wine	Mineral	Cattle	Oil	Gas	Coal
*x* = 6	*x* = 5	*x* = 7	*x* = 8	*x* = 3	*x* = 2	*x* = 4	*x* = 9	*x* = 1
*a*_1_ = 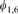	*a*_2_ = *a*_1_+ 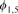	*a*_3_ = *a*_2_+ 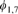	*a*_4_ = *a*_3_+ 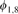	*a*_5_ = *a*_4_+ 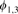	*a*_6_ = *a*_5_+ 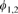	*a*_7_ = *a*_6_+ 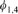	*a*_8_ = *a*_7_+ 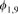	*a*_9_ = *a*_8_+ 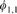
0.909	1.709	2.509	4.009	4.676	5.676	6.676	7.676	9.009
*b*_9_ = *b*_8_+ 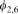	*b*_8_ = *b*_7_+ 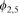	*b*_7_ = *b*_6_+ 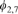	*b*_6_ = *b*_5_+ 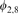	*b*_5_ = *b*_4_+ 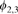	*b*_4_ = *b*_3_+ 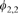	*b*_3_ = *b*_2_+ 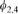	*b*_2_ = *b*_1_+ 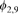	*b*_1_ = 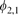
9.008	8.099	7.299	6.499	4.999	4.332	3.332	2.332	1.332

It is assumed that Villages 1 and 2 have goods in their initial possession list with a difference in sum of *utilities* for initial goods of possession. If one village has a final *utility* sum that is lower than the initial total *utility* value, then the product of utility gain is negative. This usually occurs when one village has a significantly larger possession list in comparison with the other one. Let us consider the case of having a larger difference in sum of *utilities* for initial goods. Assume that Villages 1 and 2 have different goods as shown by Table 4.

**Table 4:** *Utility* of goods for Villages (*i* 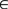
{1, 2}).

Village	*x*	Goods	*u_1,x_*	*u_2,x_*
1	1	Coal	2	4
	2	Cattle	2	2
	3	Mineral	2	1
	4	Oil	2	2
	5	Salt	4	1
	6	Iron	10	1
	7	Steel	4	1
2	8	Wine	6	2
	9	Gas	2	2

The initial values of the utility sums for the villages are
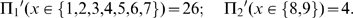
(15)Bargaining, based on the assumption that villages have initial possession lists, results in
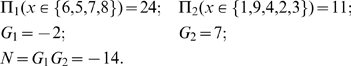
(16)The proposed approach can present the *utility* gains with optimality by assigning of goods *a priori*. Referring to the *priority* ordering, and listing the cumulative *utility* values for Villages 1 and 2, some of the goods can be assigned *a priori* in Table 5 before determination of 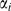 using Table 6.

**Table 5:** Cumulative *utilities* of goods for Villages (*i* 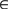
{1,2}).

Goods assigned to Village 1					Goods assigned to Village 2			
Iron	Salt	Steel	Wine	Mineral	Cattle	Oil	Gas	Coal
*x* = 6	*x* = 5	*x* = 7	*x* = 8	*x* = 3	*x* = 2	*x* = 4	*x* = 9	*x* = 1
*a*_1_ = *u*_1,6_	*a*_2_ = *a*_1_+*u*_1,5_	*a*_3_ = *a*_2_+*u*_1,7_	*a*_4_ = *a*_3_+*u*_1,8_	*a*_5_ = *a*_4_+*u*_1,3_	*a*_6_ = *a*_5_+*u*_1,2_	*a*_7_ = *a*_6_+*u*_1,4_	*a*_8_ = *a*_7_+*u*_1,9_	*a*_9_ = *a*_8_+*u*_1,1_
10	14	18	24	26	28	30	32	34
*b*_9_ = *b*_8_+*u*_2,6_	*b*_8_ = *b*_7_+*u*_2,5_	*b*_7_ = *b*_6_+*u*_2,7_	*b*_6_ = *b*_5_+*u*_2,8_	*b*_5_ = *b*_4_+*u*_2,3_	*b*_4_ = *b*_3_+*u*_2,2_	*b*_3_ = *b*_2_+*u*_2,4_	*b*_2_ = *b*_1_+*u*_2,9_	*b*_1_ = *u*_2,1_
16	15	14	13	11	10	8	6	4

**Table 6:** Cumulative *utility-distance products* of unassigned goods.

Goods		
Cattle	Oil	Gas
*x* = 2	*x* = 4	*x* = 9
*a*_1_ = 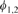	*a*_2_ = *a*_1_+ 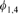	*a*_3_ = *a*_2_+ 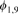
1.000	2.000	3.000
*b*_3_ = *b*_2_+ 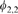	*b*_2_ = *b*_1_+ 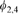	*b*_1_ = 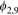
3.000	2.000	1.000

Here, a possession list that pertains to Village 1 comprises of Iron, Salt, Steel, Wine, and Mineral, while that of Village 2 consists of Coal only. The remaining goods are Cattle, Oil, and Gas. These goods can be assigned by determining the *utility-distance products* for Village 1 and 2 to give 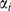 as Oil. The *utilities* and gains are
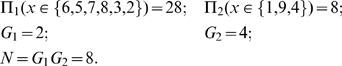
(17)In practice, we create an initial list of goods and then assign the remaining ones. This can save computation of the *utility-distance products* for the goods of bargain that can be initialized *a priori*.

### 3.3 Three-player Bargaining Game (*n* = 3)

The geometrical framework for a bargaining game by two players can be generalized to *n* players. In general, this method facilitates an ordered *priority* list for every player when determining the equilibrium point. This concept can be extended to the calculation of bargaining solution for multiple villages involved in the exchange of their produce limited by our assumptions. As only one village needs to be considered at a time for good assignment, we are able to determine the assignment of bargaining goods towards attaining EP and maximization of gains.

**Figure 5:**
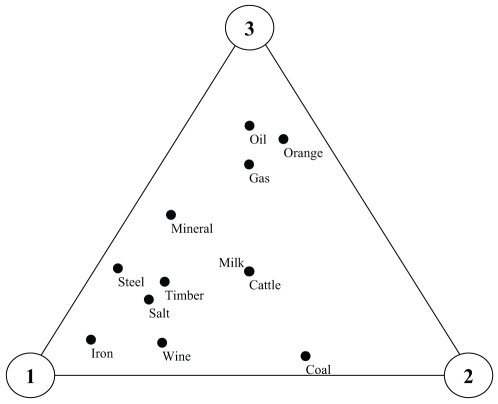
Geometrical distribution of goods for Villages (*i* 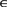
{1,2,3}).

**Figure 6:**
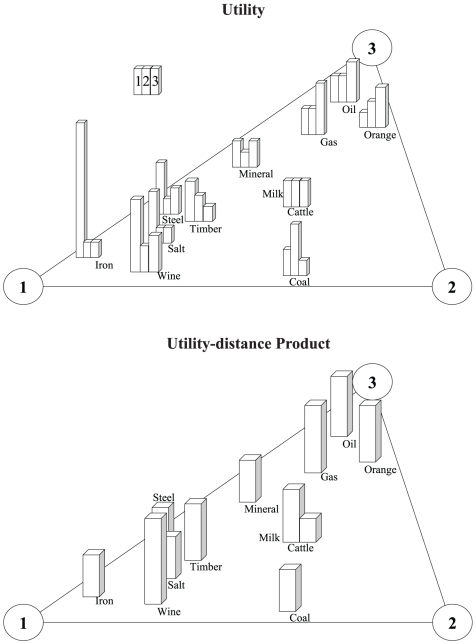
Geometrical distribution of goods presented with magnitudes of *utility* and *utility-distance product* for Villages (*i* 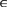
{1,2,3}).

Suppose that three villages (*i* 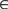
{1,2,3}) is involved in a bargaining game (*n* = 3). The *normalized distances* and *utility-distance products* of initially possessed goods are presented in Table 7. The geometrical representation of the goods is illustrated in Figure 5, which shows the *utility* values of the goods for each village spatially on a two-dimensional plane. Figure 6 shows the *utility-distance product* of each item to the respective player. The goods are positioned at various loci of the enclosed triangle. Here, the locus is based on the distances from item to each player vertex.

**Table 7:** *Utility-distance product* of goods for Villages (*i* 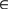
{1,2,3}).

Village	*x*	Goods	*u_1,x_*	*d_1,x_*	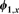	*u_2,x_*	*d_2,x_*	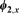	*u_3,x_*	*d_3,x_*	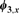
1	1	Coal	2	0.286	0.572	4	0.143	0.572	1	0.572	0.572
	2	Cattle	2	0.333	0.666	2	0.333	0.666	2	0.333	0.666
	3	Mineral	2	0.250	0.5	1	0.500	0.500	2	0.250	0.500
	4	Oil	2	0.429	0.858	2	0.429	0.858	3	0.143	0.858
	5	Salt	4	0.111	0.444	1	0.444	0.444	1	0.444	0.444
2	6	Iron	10	0.0476	0.476	1	0.476	0.476	1	0.476	0.476
	7	Steel	4	0.143	0.572	1	0.572	0.572	2	0.286	0.572
	8	Wine	6	0.167	1.002	2	0.500	1.000	3	0.333	0.999
	9	Gas	2	0.400	0.8	2	0.400	0.800	4	0.200	0.800
3	10	Orange	1	0.545	0.545	2	0.273	0.546	3	0.182	0.546
	11	Timber	3	0.182	0.546	2	0.273	0.546	1	0.545	0.545
	12	Milk	1	0.333	0.333	1	0.333	0.333	1	0.333	0.333

The initial values of the utility sums for village 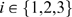 are
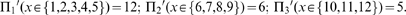
(18)An equilibrium condition can be determined by
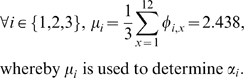
(19)From Table 8, we deduced that 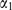 = Wine, 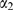 = Milk, and 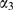 = Steel. Here, goods assigned to Villages 1, 2, and 3 are {Iron, Salt, Steel, and Wine}, {Coal, Orange, Timber, Cattle, and Milk}, and {Oil, Orange, Gas, Mineral, and Steel} respectively. The Orange entity is assigned simultaneously to Village 2 and Village 3's possession lists. And Steel appears in Village 1 and Village 3's possessions. Since Steel has a higher *priority* to Village 1 than Village 3, and the same condition occurs for the case of Village 2 versus Village 3, during the assignment of Orange, Village 1 and Village 3 are assigned Steel and Orange respectively.

**Table 8:** Cumulative *utility-distance products* of goods for Villages (*i* 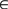
{1,2,3}).

Village (*i* = 1)											
Iron	Salt	Steel	Wine	Timber	Mineral	Coal	Cattle	Milk	Gas	Oil	Orange
*x* = 6	*x* = 5	*x* = 7	*x* = 8	*x* = 11	*x* = 3	*x* = 1	*x* = 2	*x* = 12	*x* = 9	*x* = 4	*x* = 10
*a*_1_ = 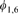	*a*_2_ = *a*_1_+ 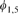	*a*_3_ = *a*_2_+ 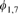	*a*_4_ = *a*_3_+ 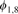	*a*_5_ = *a*_4_+ 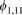	*a*_6_ = *a*_5_+ 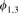	*a*_7_ = *a*_6_+ 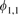	*a*_8_ = *a*_7_+ 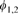	*a*_9_ = *a*_8_+ 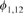	*a*_9_ = *a*_8_+ 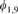	*a*_9_ = *a*_8_+ 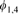	*a*_9_ = *a*_8_+ 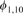
0.476	0.920	1.492	2.494	3.040	3.540	4.112	4.778	5.111	5.911	6.769	7.314

The result of assignment is displayed in Table 9. Here, we obtain
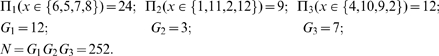
(20)The geometrical approach in predicting the Pareto-optimal solution set for multiple players (*n* = 3) demonstrates that equilibrium can be attained by balancing the *utility-distance product* values of all goods among the three villages. Payoff in terms of *utility* is arbitrarily set to present a barter trade scenario here as a case study. In practice, the game is based on an arbitrary number of players in order to demonstrate the fundamental principles of the technique without incurring further complexities that may arise if more players are used.

**Table 9:** Goods assignment for Villages (*i* 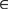
{1,2,3}).

Village	Goods			
1	Iron	Salt	Steel	Wine
	*x* = 6	*x* = 5	*x* = 7	*x* = 8
2	Coal	Timber	Cattle	Milk
	*x* = 1	*x* = 11	*x* = 2	*x* = 12
3	Oil	Orange	Gas	Mineral
	*x* = 4	*x* = 10	*x* = 9	*x* = 2

## Discussion

The solution to a bargaining problem is reflected as the Pareto-optimal EP of the set of bargaining alternatives. The combination of possible sets escalates when the number of strategy choices and players increases. To reduce the number of alternatives considered in a combinatorial set, the concept of using spatial distance to represent the significance of the item to the player in a non-zero-sum game can be implemented.

To allow assignment of items while considering only one player at a time, the concept of using the geometrical distance to represent *utility* of items is introduced. Then, this technique can extract a smaller set of alternatives from the super set of strategy alternatives and enables the Pareto-optimal EP to be obtained from this reduced set. The attainment of the solution is determined by the nature of the strategy choices. Instead of using multivariate polynomial functions, the geometrical approach reduces the computational expenses involved in determining EP. For instance, the concept of shifting the bargaining outcome towards Pareto-optimal equilibrium is by geometrically positioning items of higher *priority* to one player with respect to the others using a shorter relative distance.

Spatial representation of items based on their *utility* can be used to derive the Pareto-optimal EP in non-zero-sum games. This method relies on the concept of spatial distribution of items respective to its level of significance to the players involved. The efficiency of calculating the EP for *n*- players in a game has been greatly improved, but there are a few limitations that have yet to be resolved such as the definition of an optimality equation in the bargaining game. This is a very important tool as the EP of a large-scale game is the main objective of almost all the real games in the world.
